# Alcohol consumption, hypertension and obesity: Relationship patterns along different age groups in Uganda

**DOI:** 10.1016/j.pmedr.2020.101141

**Published:** 2020-06-13

**Authors:** Nazarius Mbona Tumwesigye, Gerald Mutungi, Silver Bahendeka, Ronald Wesonga, Agaba Katureebe, Claire Biribawa, David Guwatudde

**Affiliations:** a Department of Epidemiology and Biostatistics, Makerere University School of Public Health, Uganda; b Control of Non-communicable Diseases Desk, Ministry of Health, Kampala, Uganda; c Department of Internal Medicine St. Francis Hospital Nsambya, Kampala, Uganda; d School of Statistics and Planning, Makerere University, Kampala, Uganda

**Keywords:** Hypertension, Obesity, Overweight, Non-communicable diseases, Alcohol, Alcohol consumption, Alcohol epidemiology, Disease across age groups, Life span

## Abstract

The prevalence of non-communicable diseases including hypertension and obesity is rising and alcohol consumption is a predisposing factor. This study explored the effect of alcohol consumption patterns on the hypertension-age group and obesity-age group relationships.

The data were extracted from the 2014 National NCD Survey of adults aged 18–69 years. Hypertension was defined as a condition of having systolic blood pressure ≥140 mmHg or diastolic blood pressure ≥90 mmHg while obesity was defined as having a body mass index ≥30 kg/m^2^. Frequent alcohol consumption was measured as alcohol use ≥3 times a week. Multivariable log binomial regression analysis was used to assess independent relationship between the outcomes and alcohol consumption.

The prevalences of hypertension, frequent alcohol consumption and obesity increased across age groups but were divergent towards last age group. Hypertension prevalence ratios were higher with higher age groups among moderate and nondrinkers but not among frequent drinkers. Alcohol drinking pattern modified the age hypertension relationship in a model with ungrouped age. The drinking pattern did not modify obesity-age relationship.

Alcohol consumption pattern appeared to modify the hypertension-age group relationship. However, more research is needed to explain why prevalence ratios are higher with higher age groups among moderate drinkers and abstainers while they stagnate among the frequent drinkers. There was no evidence to show the effect of alcohol consumption on obesity-age group relationship.

## Highlights

• Hypertension prevalence ratios don’t rise with age groups among frequent drinkers.
• Alcohol consumption pattern partly modifies the age-hypertension relationship.
• Alcohol consumption pattern does not modify obesity-age relationship.

## 1 Introduction

Alcohol consumption is widely known to be associated with high blood pressure and obesity (Musinguzi and Nuwaha, 2013, Wamala, 2009, WHO, 2009) both of which are also known to increase with age (Wamala, 2009, Lloyd-Jones et al., 2005, Kopelman, 2000). However, little is known on how alcohol affects high blood pressure and obesity trajectories across the age spectrum. So far studies have reported varying effects of alcohol on the hypertension and age groups relationship. For example, a study in the USA, found that in young population aged 18–26 blood pressure reduced among those who took 2–3 drinks a day but rose higher with more or less alcohol intake (Gillman, 1995) while in a study in Germany it was found that there was a linear relationship between alcohol intake and blood pressure for only men aged 20–34 and 50–74 and women aged above 49 years (Fortmann, 1983). Variations in alcohol intake-blood pressure-age relationships have also been established in France (Milon, 1982), Japan (Wakabayashi and Araki, 2010), South Africa (Zatu, 2014), Ghana (Sanuade et al., 2018) and Netherlands (van Leer et al., 1994). Such varying evidence calls for more localized research that can inform local intervention. Other factors known to have an association with hypertension and can have a potential confounding effect on the hypertension-age relationship include physical activity (Sobngwi, 2002, Agyemang et al., 2006), sex (Twagirumukiza, 2011), education level (Peltzer and Phaswana-Mafuya, 2013), occupation (Leng, 2015) and marital status (Daniel, 2013).

Like hypertension-age relationship, obesity-age relationship is affected by alcohol intake differently in different studies (Xu, 2016). Obesity is known to increase with age to a certain level, mostly around 60–70 years, and reduce but this depends on many factors (Agyemang, 2016, Shayo and Mugusi, 2011) including alcohol consumption. A study in Japan found that alcohol consumption reduced the odds of obesity in younger (below median age) men but the relationship was not prominent among older men (Wakabayashi, 2011). Some prospective studies show that light to moderate alcohol consumption does not increase obesity but heavy alcohol consumption does (Traversy and Chaput, 2015). Work published in 2018 showed that women in England and Scotland that abstained from alcohol had higher odds of obesity compared to those that drank alcohol (O’Donovan et al., 2018) thus showing potential modifying effect of alcohol consumption on age and obesity relationship. Differing effect of alcohol on the obesity- age relationship has also been found in studies in South Africa (Sogunle, 2018) and Ghana (Poku, 2011). Owing to a strong obesity and hypertension association several factors are associated to both outcome variables. Several studies have shown a strong association between obesity and marital status (Fitzgibbons Shafer, 2010), sex (Lundeen, 2016), education (Neupane et al., 2015), occupation (Scott, 2013) and physical activity (Muthuri, 2014) which are also associated with hypertension. Therefore, the same factors are potential confounders for the obesity-age relationship.

In Uganda, 7% of women and 1% of men in age group 15–49 are obese (UBOS and ICF, 2016). In 2014, the National Non-Communicable Diseases (NCD) Risk Factor Survey found that the prevalence of hypertension was 26.4% and it was associated with higher Body Mass Index (>25 kg/m^2^) (MOH, 2014, Guwatudde, 2015). On alcohol consumption, the country is now rated 5th highest consumer in Africa in terms of per capita pure alcohol with an estimated average consumption of 9.8 L (14.4 L for men and 5.2 L for women) of pure alcohol per person per year (WHO, 2018). There is a paucity of research on effect of alcohol consumption on the obesity-age group and hypertension-age group relationships (Klatsky and Gunderson, 2008, Stranges, 2004) especially in developing countries yet this would inform age group specific intervention. This study adds an effort on reducing this knowledge gap.

## 2 Materials and methods

### 2.1 Design of the study

This paper uses secondary data from the National Non-Communicable Disease (NCD) Risk Factor Survey of 2014. In the survey 3,987 participants were interviewed but only 3906 had 3 blood pressure measurements. The data were collected using the STEPwise approach to surveillance (STEPS). STEPS is a World Health Organization method that provides a standardized method for collection, analysis and dissemination on risk factors for non-communicable diseases (NCD) (MOH, 2014). The approach uses a multi-stage cluster sampling design to obtain a nationally representative sample. The household response rate for the study was 99%. More details on the methods used to collect data, individual response rates and the distribution of the respondents by all background characteristics can be obtained from one published paper (Guwatudde, 2016)and the national NCD report (MOH, 2014).

For purposes of this study, only relevant variables were provided by the management of the NCD survey. Key among these variables were frequency of alcohol consumption in the past 12 months, height, weight, biometrics that include hypertension and body mass index, and socio-demographic-economic characteristics of the respondents. The key dependent variables were having hypertension and having obesity while the key independent variable was age or age group. Alcohol consumption was a modifying effect/interaction variable and it’s another variable of focus. The rest of the variables were mere control variables that were included in the multivariable models depending on their known confounding effects. These include gender, marital status, occupation, physical activity, income level and education.

### 2.2 Measurements

Hypertension was defined as systolic blood pressure ≥ 140 mmHg or diastolic blood pressure ≥ 90 mmHg (WHO, 2005). The average of the last two blood pressure readings was used in analysis. This is consistent with the procedures used in the main publication from the original study (Guwatudde, 2015). The blood pressure measurements were taken on the left arm with the participant in the sitting position using battery powered digital blood pressure machine (Boso Medicus Uno). This device is among those approved by Germany hypertension league quality seal protocol for blood pressure-measuring devices (Tholl, 2016).

Obesity was defined as body mass index greater than or equal to 30 kg/m^2^ as per WHO recommendations (Obesity, 2019). The body mass index (BMI) is computed as weight of an individual divided by the square of his/her height. Standing height was preferred and it was measured with a laser stadiometer incorporated in the compact SECA 877 (Schein, 2020) that included a pre-calibrated digital weighing scale. In event of failure of the laser sensor stadiometer height would be manually measured with the participant standing upright against a wall on which a height mark was made, and a tape measure used to derive the height (Guwatudde, 2015). Measurements were taken with the participant in barefoot, in light clothing, standing with the back and head against the wall, with heels together. The participant was asked to stretch to the fullest. Weight measurements were taken using a pre-calibrated digital weighing scale (SECA 877). The height was measured to the nearest centimetre while weight was measured to the nearest tenth of a kilogram. Data collectors were trained on how to take all these physical measurements by World Health Organization and Ministry of Health Officials.

To measure alcohol consumption, we used a question on how frequently the respondent had had at least one standard alcoholic drink during the previous 12 months. The responses were daily, 5–6 days per week, 3–4 days per week, 1–2 days per week, 1–3 days per week and less than once a month. For this paper the response from this question was recoded as ≥3 times (frequent), <3 days (moderate) and 0 (abstainer). Before this frequency question there was one on experience of alcohol consumption as “have you ever consumed any alcohol such as beer, wine, spirits, or waragi, malwa, or any local brew (Yes/NO)?

Physical activity (PA) was measured using the question on how many days the respondent does vigorous- intensity or moderate-intensity activities as part of his/her work, travel and recreational exercises. Each of these questions was followed by a question on how many hours the activity took in hours and minutes. This enabled computation of the total number of minutes the respondent took on moderate and vigorous intensity activities. WHO recommends that throughout a week, including activity for work, during transport and leisure time, adults should do at least 150 min of moderate-intensity or 75 min of vigorous-intensity physical activity or an equivalent combination of moderate and vigorous-intensity physical activity achieving at least 600 Metabolic Equivalent Task (MET)-minutes (WHO, 2020). With this guideline the total number of minutes on the moderate or intensity activities were recoded as 1 for having the WHO minimum recommended PA (≥150 for moderate or ≥ 75 for vigorous) and 0 for not having achieved the minimum required.

In the report and paper produced from the same data set the definition for hypertensive covered those who reported being on regular anti-hypertensive therapy (Guwatudde, 2015) but the data set used on this paper does not include information on whether a person was on treatment.

The key analytical outputs were prevalence, crude prevalence ratios and adjusted prevalence ratios. Crude prevalence ratio for an age group was computed as the prevalence of hypertension or obesity in that age group divided by prevalence in the base age group which was mostly 18–19 years. This prevalence ratio got adjusted after controlling for the confounding factors and frequency of alcohol consumption in a multivariable model. Computations for prevalence and prevalence ratios were carried out by sex in some tables and chart to highlight gender differences.

### 2.3 Statistical analysis

We used log binomial models to establish factors associated with diabetes and hypertension across the age groups. In the log binomial models we model relative risks or prevalence ratios directly. This is in contrast with binomial logistic regression where log odds of occurrence of an event are modelled. Unlike logistic regression log binomial models do not overestimate their coefficients when the outcome of interest is a common occurrence (Martinez, 2017, Blizzard and Hosmer, 2006). Modified Poisson regression could have been another choice of analysis technique but its efficiency is limited when some categories have small observations (Chen, 2018). We used Stata V14 software (StataCorp LLC, College Station, TX, USA) to analyse data which were weighted using a sampling weight variable. STATA’s *svyset* command was used to weight the data before analysis.

Charts were used to show trends of prevalence ratio for hypertension and obesity across age groups and effect of alcohol consumption. A non-parametric test, Wilcoxon’s sign rank test, was used to test the significance of a difference in prevalence ratios between any two categories (Nahm, 2016). This test was preferred because the observations were few and they were not normally distributed which is a condition for use of parametric tests like T-test.

## 3 Results

### 3.1 Characteristics of respondents

The respondents were aged 18–69 years with 60% of them being women and 74% within the age range 21–50 years. The age distribution did not differ by gender (Table 1). Two thirds were married or in relationship, but relationship status varied by gender. Two fifths had attained primary school, but among women a higher proportion did not have any formal education. Nearly two thirds were employed, but a higher percentage of men were employed compared to the women. The median income per month was UgShs 100,000(≈USD 30) and it was significantly higher among men (Ug Shs 110,000) (USD ≈33) than women (Ug Shs 60,000) (≈USD 18).

**Table 1:** Background characteristics of the respondents in NCD Survey, 2014 Uganda.

Characteristics			All n (%)	Chi-sq. test p-value
	Men n (%)	Women n (%)		
Age				
18–19	124 (7.9)	181 (7.8)	305 (7.8)	
20–24	258 (16.4)	362 (15.5)	620 (15.9)	
25–29	261 (16.6)	398 (17.0)	659 (16.9)	
30–34	210 (13.4)	331 (14.2)	541 (13.9)	
35–39	195 (12.4)	275 (11.8)	470 (12.0)	NS-
40–44	158 (10.1)	199 (8.5)	357 (9.1)	p > 0.05
45–49	103 (6.6)	173 (7.4)	276 (7.1)	
50–54	108 (6.9)	157 (6.7)	265 (6.8)	
55–59	66 (4.2)	79 (3.4)	145 (3.7)	
60–64	49 (3.1)	99 (4.2)	148 (3.8)	
65–69	38 (2.4)	82 (3.5)	120 (3.1)	
Marital status^a^				
Single	385 (24.5)	229 (9.8)	614 (15.7)	
Married/in relationship	1045 (66.6)	1552 (66.5)	2597 (66.5)	
Widowed/Separated/divorced	140 (8.9)	552 (23.7)	692 (17.7)	p < 0.001
Education^b^				
None	118 (7.6)	521 (22.3)	639 (16.4)	
Primary	630 (40.4)	973 (41.7)	1603 (41.2)	
Secondary+	810 (52.0)	839 (36.0)	1649 (42.4)	p < 0.001
Work status				
Employed	1192 (75.9)	1357 (58.1)	2549 (65.3)	
Student/unpaid worker	305 (19.4)	816 (34.9)	1121 (28.7)	
Unemployed	73 (4.7)	163 (7.0)	236 (6.0)	p < 0.001
Income per month-median (IQR)	110,000 (40,000–300,000)	60,000 (20,000–200,000)	100,000 (20,000–200,000)	p < 0.01
Drinks alcohol 3+ times a week	246 (15.7)	79 (3.4)	325 (8.4)	p < 0.001
Obese (body mass index ≥ 30 kg/m^2^)	34 (2.2)	200 (9.4)	234 (6.4)	p < 0.001
Has hypertension (Systolic ≥ 140 mmHg/diastolic ≥ 90 mmHg)^c^	411 (26.2)	550 (23.5)	961 (24.6)	NS-p > 0.05

Met WHO recommendation for physical activity	1500 (95.5)	2185 (93.5)	3685 (94.3)	p = 0.008

All	1570 (100.0)	2336 (100.0)	3906 (100.0)	

^a^3 did not provide information ^b^15 refused to provide information ^c^The persons with hypertension don’t include those who had normal blood pressure (BP) but were taking BP drugs at the time of visit. NS- Not significant at 5% level.

The level of frequent alcohol consumption (3 or more times a week) was 8% but it was significantly higher among men (16%) than women (3%). Obesity was at 6% and it was higher among women (9%) than men (2%). Hypertension level was at 25% and it did not significantly differ by gender.

### 3.2 Alcohol consumption by background characteristics

The prevalence of frequent alcohol consumption increased by age group among men but not as much as among women (Table 2). The trend of frequent alcohol consumption across age group was also significant in both men and women (p < 0.001). Frequent alcohol consumption was most common among the widowed/separated (9.4%) and those without formal education (10.8%) but the pattern was more evident among men. Overall, frequent alcohol consumption did not significantly vary by work status but varied by obesity status and hypertension status. Those who were hypertensive had a significantly higher frequency of alcohol consumption than those who were not (p < 0.05).

**Table 2:** Prevalence of frequent alcohol consumption by background characteristics, NCD survey 2014, Uganda.

*Background characteristics*	Gender		All n (%)
	Men n (%)	Women n (%)	
Age	***	***	***
18–19	4 (3.2)	1 (0.6)	5 (1.6)
20–24	19 (7.5)	9 (2.5)	28 (4.6)
25–29	30 (11.6)	5 (1.3)	35 (5.3)
30–34	30 (14.3)	10 (3.0)	40 (7.4)
35–39	40 (20.6)	10 (3.6)	50 (10.7)
40–44	37 (23.4)	9 (4.5)	46 (12.9)
45–49	24 (23.5)	9 (5.2)	33 (12.0)
50–54	23 (21.3)	6 (3.8)	29 (10.9)
55–59	17 (25.8)	3 (3.8)	20 (13.8)
60–64	14 (28.6)	8 (8.1)	22 (14.9)
65–69	8 (21.1)	9 (11.1)	17 (14.3)
Marital status^a^	*	*	*
Single	29 (7.4)	8 (3.5)	37 (6.0)
Married/in relationship	185 (17.5)	44 (2.8)	229 (8.7)
Widowed/Separated/divorced	38 (26.1)	29 (5.1)	67 (9.4)
Education^b^	***	***	**
None	26 (22.2)	43 (8.3)	69 (10.8)
Primary	120 (19.1)	25 (2.6)	145 (9.1)
Secondary+	97 (12.0)	11 (1.3)	108 (6.6)
Work status			
Employed	191 (16.1)	37 (2.7)	228 (9.0)
Student/unpaid worker	41 (13.6)	34 (4.2)	75 (6.7)
Unemployed	14 (19.2)	8 (5.0)	22 (9.4)
Obese			**
Yes	5 (14.7)	3 (1.5)	8 (3.4)
No	239 (15.7)	69 (3.6)	308 (9.0)
Hypertensive^c^	*		**
Yes	77 (18.9)	25 (4.6)	102 (10.7)
No	169 (14.6)	54 (3.0)	223 (7.6)
Met WHO recommendation for physical activity			*
Yes	231 (15.5)	74 (3.4)	305 (8.3)
No	15 (21.4)	5 (3.3)	20 (9.1)

All	247 (15.7)	79 (3.4)	325 (8.4)

^a^3 did not provide information ^b^15 refused to provide information ^c^ The persons with hypertension don’t include those who had normal blood pressure (BP) but were taking BP drugs at the time of visit NS- Not significant at 5% level. Chi-sq p-values: * p ≤ 0.05 **p < 0.01 ***p < 0.001.

### 3.3 Alcohol consumption, hypertension and frequent alcohol consumption across different age groups

**Fig. 1:**
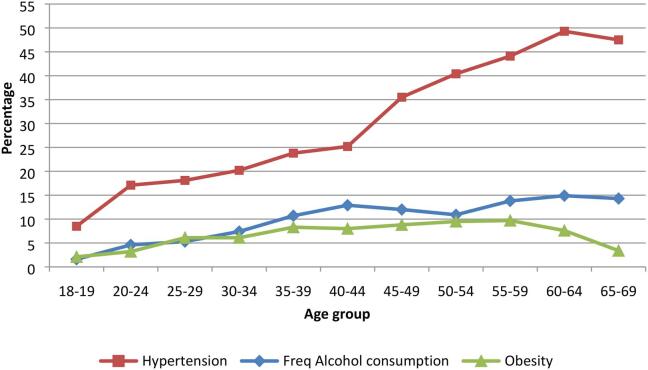
Levels of hypertension, obesity and frequent alcohol consumption across different age groups- NCD survey 2014, Uganda.

Fig. 1 shows that the levels of hypertension were higher among older people from 8.5% in the 18–19 age group to 47.8% in the age group 65–69. The prevalence of frequent alcohol consumption was higher with higher age groups (1.6% in 18–19 to 14.3% in 65–69) but at a slightly higher pace than obesity levels (from 2.1% in 18–19 to 9.7 in 55–59). Values for all the three indicators start at the same level and sharply diverge after 40–44 age group. A significant test of the gradients of the charts showed significance (p < 0.01) for hypertension but not for alcohol consumption and obesity (p > 0.2).

### 3.4 Hypertension by different age groups after controlling for alcohol consumption and other key factors

The prevalence ratios for hypertension rose by age group and this persisted after controlling for frequency of alcohol consumption and other key factors (Table 3). A test of significance of an interaction between frequency of alcohol consumption, hypertension and age group did not yield any significance at 5% level but when age group was replaced with age in single years it was significant. This shows that the relationship between alcohol consumption and hypertension significantly changed by single year rather than by age group. The table further shows that hypertension level is highest (31%) among the frequent drinkers compared to the moderate drinkers (26%) and abstainers (23%) and this may help explain the effect of alcohol consumption pattern on the age-hypertension relationship in further analysis.

**Table 3:** Hypertension Prevalence ratios along different age groups and other factors, NCD survey 2014, Uganda.

Characteristics	Prevalence n (%)	Un-adjusted Prevalence ratios UPR (95%CI)	Adjusted Prevalence ratios APR (95%CI)
Age			
18–19	26 (8.5)	1.0	
20–24	106 (17.1)	1.46 (1.03–2.07)*	2.16 (1.43–3.26)***
25–29	119 (18.1)	1.64 (1.17–2.30)**	2.37 (1.57–3.59)**
30–34	109 (20.2)	1.80 (1.28–2.53) **	2.82 (1.85–4.31)***
35–39	112 (23.8)	2.05 (1.46–2.88) ***	3.32 (2.17–5.08) ***
40–44	90 (25.2)	2.54 (1.81–3.57) ***	3.59 (2.32–5.53) ***
45–49	98 (35.5)	3.02 (2.15–4.24) ***	5.01 (3.27–7.68) ***
50–54	107 (40.4)	3.55 (2.54–4.94) ***	5.90 (3.87–9.01) ***
55–59	64 (44.1)	3.87 (2.73–5.48) ***	6.22 (4.02–9.64) ***
60–64	73 (49.3)	4.29 (3.05–6.02) ***	7.27 (4.73–11.19) ***
65–69	57 (47.5)	4.26 (3.01–6.03) ***	7.09 (4.56–11.02) ***
Frequency of alcohol consumption in past 12 months			
3+ times weekly	102 (31.4)	1	1
<3 times weekly	261 (25.8)	0.90 (0.75–1.08)	0.99 (0.81–1.19)
No/doesn’t drink	594 (23.3)	0.79 (0.67–0.94)**	0.95 (0.79–1.13)
Gender			
Men	411 (26.2)	1	1
Women	550 (23.5)	0.92 (0.83–1.02)	1.01 (0.89–1.14)
Marital status			
Single	120 (19.5)	1.0	1
Married/in relationship	617 (23.8)	1.25 (1.05–1.47)*	0.73 (0.60–0.89)**
Widowed/Separated/divorced	223 (32.2)	1.67 (1.39–2.01)**	0.76 (0.60–0.95)*
Work status			
Employed	102 (23.4)	1.0	1
Self employed	547 (25.3)	0.86 (0.70–1.06)	0.95 (0.79–1.14)
Student/unpaid worker	248 (22.1)	0.76 (0.60–0.95)*	0.89 (0.73–1.10)
Unemployed	64 (27.1)	0.96 (0.70–1.31)	0.94 (0.72–1.23)
Education†			
None	180 (28.2)	1.0	
Primary	383 (23.9)	0.87 (0.77–0.99)*	1.07 (0.92–1.24)
Secondary+	390 (23.7)	1.12 (0.92–1.37)	1.19 (1.02–1.40)*
Average Income per month (Ug Shs)			
<=500,000	236 (25.4)	1	
>500,000	21 (31.3)	1.05 (0.71–1.56)	–
Met WHO recommendation for physical activity			
No	65 (29.4)	1	1
Yes	896 (24.3)	0.83 (0.67–1.02)	0.95 (0.77–1.18)

Age group*Frequency of alcohol consumption interaction	M	M	M

†15 refused to provide information. – left out because of very little contribution to model fitness M = many levels. The model has been adjusted for gender, marital status, occupation, physical activity and education. Wald’s test for interaction between age group and frequency of alcohol consumption yielded p = 0.13.while that for age in single year and alcohol consumption yielded p = 0.02. Wald’s test p-values:* p < 0.05 ** p < 0.01 *** p < 0.001.

**Fig. 2:**
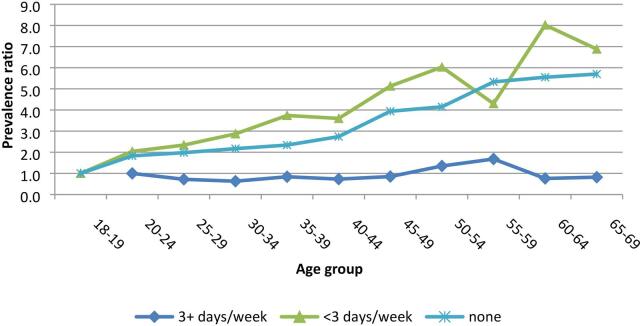
Prevalence ratios for Hypertension across age groups by frequency of alcohol consumption among both men and women, NCD survey 2014, Uganda.

Fig. 2 shows that the prevalence ratios across age groups among those who drank most frequently stagnated while among those who drank moderately and those who never drank the ratios were higher with higher age groups. It should be noted that the number of those who drank three or more times a week were fewer compared to those who never drank and those who drank less than 3 times a week.

**Fig. 3:**
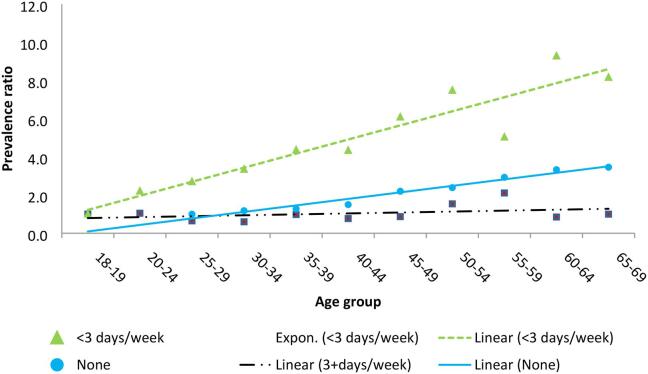
Fitted Prevalence ratios for hypertension across different age groups by alcohol consumption pattern, NCD survey 2014, Uganda NB: For non-drinkers those aged <25 years were left out because the model could not converge. The models have been adjusted for gender, marital status, occupation, physical activity and education.

Fig. 3 shows fitted trend lines for prevalence ratios for hypertension at different age groups by alcohol consumption patterns. The ratios were produced from a multivariable log binomial regression model controlling for gender, marital status, occupation, physical activity, and education. The figure shows a pattern consistent with that in Fig. 2 thus re-affirming higher risk ratios of hypertension across age groups among medium alcohol consumers (<3 days/week) and non-consumers but a negligible difference among the frequent alcohol consumers (3+ days/week). An interaction test for drinking pattern-age group term in the model did not show significance (p > 0.2) but was significant when age groups were replaced by single year age (p < 0.001).

A Wilcoxon’s sign rank test of significance between the trends for drinking 3+ days per week and <3 days per week showed a significant difference in the fitted values (p < 0.001). The same level of significance was established with comparison of the trends for drinking <3 days per week and no alcohol (abstinent). The fitted lines for medium alcohol consumers and the abstinent groups had significant gradients.

### 3.5 Obesity

**Fig. 4:**
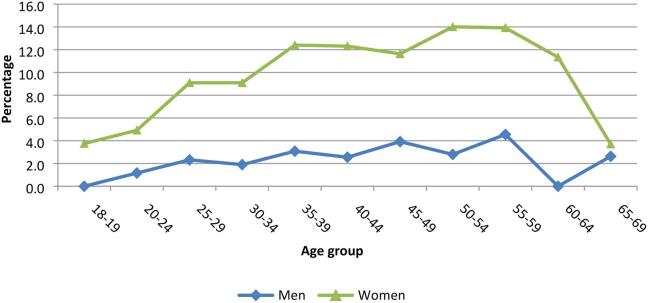
Prevalence of obesity by gender across age groups, NCD survey 2014, Uganda.

The overall prevalence of obesity was 6.4% (2.2% men, 9.4% women). The prevalence of obesity among women was lowest (3.8%) in the 18–19 age group and highest (14%) in the age group 50–54 while it was zero among men in the 18–19 age group and highest (4.6%) in the age group 55–59 (Fig. 4). The test for the difference in the two trends using Wilcoxon’s rank sum test shows a significant difference (p < 0.001).

The prevalence ratios of obesity were higher with higher age groups and the same trend was persistent after controlling for drinking patterns and other background characteristics that were significant in the bivariate models (Table 4). A Wald’s test of significance for the interaction term between age group and drinking patterns showed no significance (p > 0.2) implying that the effect of drinking pattern on obesity-age group relationship was not significant.

**Table 4:** Obesity Prevalence ratios in different age groups and other factors, *NCD survey 2014, Uganda.*

Characteristics	Prevalencen (%)	Un-adjusted Prevalence ratio UPR (95%CI)	Adjusted prevalence ratios APR (95%CI)
Age			
18–19	6 (2.1)	1.00	1.0
20–24	18 (3.2)	1.51 (0.61–3.77)	1.17 (0.47–2.91)
25–29	36 (6.1)	2.89 (1.23–6.79)*	1.74 (0.73–4.15)
30–34	31 (6.1)	2.89 (1.23–6.79)*	2.07 (0.86–5.03)
35–39	37 (8.3)	3.94 (1.68–9.20)**	2.76 (1.15–6.61)*
40–44	28 (8.0)	3.77 (1.58–8.97)**	2.82 (1.15–6.92) *
45–49	24 (8.8)	4.15 (1.72–9.99)**	3.06 (1.24–7.59) *
50–54	25 (9.5)	4.48 (1.87–10.75)**	3.47 (1.41–8.56) **
55–59	14 (9.7)	4.57 (1.79–11.64)**	3.82 (1.47–9.91)**
60–64	11 (7.6)	3.62 (1.36–9.58)*	2.91 (1.08–7.85)*
65–69	4 (3.4)	1.59 (0.46–5.54)	1.41 (0.40–4.97)
Sex			
Men	34 (2.2)	1	1
Women	200 (9.4)	4.33 (3.03–6.19)***	5.21 (3.53–7.69)***
Frequency of alcohol consumption in past 12 months			
3+ times weekly	8 (2.5)	1	1.00
<3 times weekly	59 (6.1)	2.41 (1.16–4.98)*	1.64 (0.76–3.52)
No/doesn’t drink	167 (7.1)	2.76 (1.37–5.56)**	1.62 (0.77–3.42)
Marital status			
Single	25 (4.0)	1	1.00
Married/in relationship	235 (8.9)	3.10 (1.77–5.41)***	1.90 (1.03–3.51)*
Widowed/separated/divorced	37 (5.2)	4.12 (2.28–7.42)***	1.84 (0.94–3.58)
Work status			
Employed	38 (10.2)	1	
Self employed	137 (6.7)	0.66 (0.47–0.93)*	0.84 (0.59–1.21)
Student/unpaid worker	41 (4.0)	0.39 (0.25–0.60)***	0.46 (0.29–0.71)**
Unemployed	18 (8.0)	0.79 (0.46–1.35)	0.84 (0.48–1.47)
Education†			
None	35 (5.7)	1.00	1.0
Primary	68 (4.5)	0.79 (0.53–1.80)	1.13 (0.76–1.69)
Secondary+	126 (8.1)	1.41 (0.98–2.03)	2.44 (1.67–3.56)***
Average Income per month (Ug Shs)			
<=500,000	55 (6.2)	1.00	–
>500,000	14 (21.2)	3.42 (2.01–5.81)***	–
Met WHO recommendation for physical activity			
No	31 (14.8)	1	1
Yes	203 (5.8)	0.40 (0.28–0.56)***	0.50 (0.36–0.71)***

Age group*alcohol consumption frequency interaction††	M	M	M

†15 refused to provide information. – left out because of very little contribution to model fitness M = Many levels †† A Wald’s test of the interaction showed p > 0.2. The model has been adjusted for gender, marital status, occupation, physical activity and education. Wald’s test p-values:* p < 0.05 ** p < 0.01 *** p < 0.001.

**Fig. 5:**
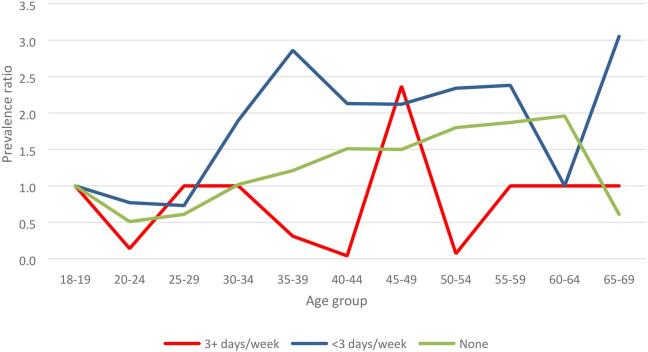
Prevalence ratios for obesity across age groups by frequency of alcohol consumption, NCD survey 2014, Uganda NB: The models have been adjusted for gender, marital status, occupation, physical activity and education.

A plot of obesity prevalence ratios over age groups derived from the multivariable model shows that the obesity prevalence ratios were higher with higher age groups, but this was evident among the abstainers and moderate drinkers only (Fig. 5). Among the moderate drinkers the ratio was highest in the age groups 35–39 (2.7) 55–59 (2.4) and 65–69 (3.9) while among the abstainers the ratio was highest in 60–64 age group (2.0) with a steady growth pattern across age groups. There was no clear pattern of relationship between obesity and age group among the frequent drinkers. The Wilcoxon’s sign rank test shows that, on average, the prevalence ratios among frequent drinkers were marginally significantly lower than those of abstainers (p = 0.04) while those among moderate drinkers did not significantly differ from those of abstainers (p = 0.12)

### 3.6 Discussion

The findings show that the prevalences of hypertension, frequent alcohol consumption and obesity were higher with higher age groups but beyond 40–44 age group the level of obesity was lower while that of frequent alcohol consumption was only slightly higher. The pattern of higher hypertension prevalence ratios with higher age groups was evident among moderate and non-drinkers but not among frequent drinkers. Alcohol drinking pattern modified a general age-hypertension relationship when age was in single years but not when it was categorised. The relationship between obesity and age group does not significantly change by alcohol drinking patterns although obesity prevalence ratios across age groups are generally higher among the abstainers and moderate drinkers compared to the frequent drinkers.

Higher levels of frequent alcohol consumption and hypertension at higher age groups are consistent with several studies in different parts of the world (Kutnikar, 2014) but at the same time inconsistent with studies in some other communities (Eigenbrodt, 2001). The increasing trend for alcohol consumption can be explained by increased access and ability to buy alcohol which may reduce later in life due to change in lifestyle, working environment and social network. Higher level of both hypertension and alcohol consumption may be explained by the threshold effect where alcohol consumption exacerbates physiological damages that may also lead to hypertension (Lip and Beevers, 1995). Higher hypertension level after 40–44 age group may be explained by the age effect (Wenger, 2018).

The variation in relationship between age and hypertension by alcohol consumption pattern has been reported by few studies (Saremi, 2004, Wu, 2019). This study has only reported a significant modifying effect of alcohol consumption on the age-hypertension relationship when age is included in the model as single years, However, non-parametric tests and graphic presentation point to a possible modifying effect of the relationship even with grouped age. The possible cause of stagnating hypertension prevalence ratios across age groups among frequent alcohol consumers could be the already high prevalence of hypertension in this category of respondents. This is consistent with other studies that monitored patterns of hypertension over years where they did not find any increase in hypertension prevalence in populations that already had high hypertension prevalence (Freeman, 1985). The smaller number of respondents in the frequent drinkers category compared to others may also have had its own effect on the results.

Larger difference in hypertension risk ratios between the older and young people among the abstainers and medium drinkers compared to the frequent drinkers was an important finding which may not be fully explained by this study. Some studies have found that non-drinkers may have higher rates of hypertension depending on their previous lifestyle (Wannamethee and Shaper, 1988). Further studies may also investigate whether frequent drinkers, who already have higher hypertension levels (31%), may be adopting lifestyles that minimise exposure to other causes of hypertension along their age spectrum. Examples of this lifestyle are walking and other forms of exercise, socialising that minimises stress levels and others.

The increase and eventual decline in obesity level with age contrasts with studies that show persistent higher obesity level at higher age groups especially among women (Al-Kandari, 2006, Aliyu, 2010) and those that show early decline in obesity level with higher age groups (Clark and Gibson, 1997, Seidell and Visscher, 2000). While some studies show higher obesity level with higher frequency of drinking (Arif and Rohrer, 2005) this study shows obesity level may possibly be lower among the frequent drinkers and higher among the moderate drinkers and abstainers. More research is needed to ascertain the effect of alcohol consumption on the obesity-age relationship.

## 4 Strengths and limitations

The strengths of this paper include being a nationwide large study that was fully designed and executed according to international standards, having evidence that has come out of a fairly exhaustive statistical analysis, and use of quality data that were collect using Personal Digital Assistants (PDAs) and had minimal non-response.

Our findings had some limitations that include the following: Firstly, the data were self-reported and as such, inherent biases or lack of knowledge about certain health conditions may have yielded an underestimate of both obesity and hypertension. Moreover, while disclosing alcohol use is not considered a sensitive matter, study participants across settings often under report actual use. Most likely these limitations yield an underestimate of the true association between alcohol use, hypertension and obesity than what has been reported in this study. Secondly, our analyses were based on a cross sectional survey and as such we could not measure the timing and prospective association between alcohol use and hypertension and obesity which would be of great importance for future prospective cohort studies. Thirdly, the number of observations for frequent drinkers was smaller than those for abstinent and moderate drinkers. This may have affected some results. In one analysis we could not split by sex because of the small number of observations. Fourthly, there are other important variables that were not available for analyses. These include medications on hypertension, dietary intake (salt, fruit and vegetable intake, processed foods) and smoking. This may have underestimated the number of hypertension cases.

## 5 Conclusion

Hypertension level was higher among those in higher age groups, but this was more evident among those aged beyond 44 years. There was some evidence, though not conclusive, that alcohol consumption pattern moderated this age-hypertension relationship. The differences in hypertension level by age group were most evident among the moderate drinkers and the abstainers while there was minimal change across the age groups among the frequent drinkers. Future research may examine this pattern more specifically and address other contextual factors not addressed in this study including potential cohort effects, age since alcohol initiation and other potential factors that can impact these findings.

The obesity levels across age groups did not differ significantly by alcohol consumption patterns. There was no clear pattern of obesity across age groups among those who were abstinent and to a large extent among those who were moderate drinkers. More research in this area will require other measures of alcohol consumption because the current measure produced a low prevalence that could have affected analysis.

While more research is needed to explain minimal variation in hypertension prevalence ratios across age groups among frequent drinkers, hypertension control programs should put more effort on moderate drinkers and abstainers. Like in other studies, results of this study have shown strong support for efforts to reduce hypertension among older persons.

Typically, while treatment protocols may include counseling patients on the risk of alcohol, it is not clear whether doctors or health care providers in Uganda have fully embraced or implemented alcohol reduction strategies such as alcohol screening and brief interventions in their treatment of hypertension. World health Organization clearly requests governments to support initiatives for screening and brief interventions for harmful use of alcohol at primary health care and other settings (WHO, 2010). However, this modification to current clinical practice should be explored and investigated further. Moreover, additional research is needed to determine the biological mechanism linking alcohol use to hypertension and what factors exacerbate this association among adults in their 40–50s. These findings underscore the importance of examining alcohol use in the context of non-communicable diseases in order to determine prevention and intervention strategies. Another area of further research is the effect of gamma-glutamyl transferase (GGT) in blood on both alcohol consumption and hypertension. Several studies show that exact mechanisms that link GGT with hypertension and alcohol abuse are not fully elucidated (Liu, 2012).

## 6 Ethics

The principal investigator obtained a written permission to use the secondary data from the management of the non-communicable diseases risk factor survey of 2014.

## CRediT authorship contribution statement

**Nazarius Mbona Tumwesigye:** Conceptualisation, methodology, validation, analysis, investigation, resources, data curation, writing draft, writing review & editing, and administration. **Gerald Mutungi:** validation and review of draft. **Silver Bahendeka:** validation and review of draft. **Ronald Wesonga:** validation and review of draft. **Agaba Katureebe:** validation and review of draft. **Claire Biribawa:** validation and review of draft. **David Guwatudde:** Conceptualization, methodology, validation, investigation, writing draft, writing review & editing.

## Declaration of Competing Interest

The authors declare that they have no known competing financial interests or personal relationships that could have appeared to influence the work reported in this paper.
